# Assessing the Allocation of Special Elderly Nursing Homes in Tokyo, Japan

**DOI:** 10.3390/ijerph14101102

**Published:** 2017-09-22

**Authors:** Ninglong You, Zhenjiang Shen, Tatsuya Nishino

**Affiliations:** School of Environmental Design, Kanazawa University, Kanazawa 920-1192, Japan; ynl49046188@gmail.com (N.Y.); tan378@se.kanazawa-u.ac.jp (T.N.)

**Keywords:** the elderly, care levels, capacity, catchment area, spatial unit, beds-needed index for SENHs (BNIS)

## Abstract

Social welfare and public health departments require reliable assessments to enhance the rationality of phased construction of special elderly nursing homes (SENHs). This paper aims to assess the allocation of SENHs based on a beds-needed index for SENHs (BNIS). This may help departments determine the priority for approving locations of SENHs more accurately with a limited budget. Traditional assessments in Tokyo use the sphere of welfare, ward, and sphere of daily life as spatial units for estimating beds-to-elderly population ratios. We calculate the BNIS by introducing a parameter-improved floating catchment area method (PI-FCA) at a smaller spatial unit, the Chome. In the PI-FCA, the catchment area is generated according to the standard of average population served by SENHs and capacity, the population demand is the population of the elderly requiring care levels 3–5 and is further modified by a coefficient of potential demand via building a multivariate linear model. Improved results were obtained using the PI-FCA. Finally, this study maps the distribution of the degree of BNIS, to provide a basis for the allocation assessment of SENHs. This caters to the needs of departments and is easily applicable in other public healthcare facilities.

## 1. Introduction

The accelerating aging of the population (65 years old or older) in Japan is more evident than other developed countries [1]. In Tokyo, overall, the number of available beds at special elderly nursing homes (SENHs) is far less than the elderly population in need [2]. It has been such a problem that the Departments of Social Welfare and Public Health (departments, for short) in Tokyo Metropolitan developed the Senior Citizen Health and Welfare Programs (programs, for short) to push the construction of SENHs at the relative shortage sphere of welfare, ward, and sphere of daily life. These programs, serving as guidance for phased construction of SENHs, were formulated from 2006 and reviewed every three years. In order to determine the priority for approving the locations of SENHs in short-term construction more accurately, departments need reliable assessments for the accurate allocation of SENHs. However, related planning based on present assessments do not have enough guiding significance to provide effective information for departments and social welfare corporations. Therefore, this paper focuses on improving assessments for the allocation of SENHs in programs, proposes a beds-needed index for SENHs (BNIS), and maps the distribution of the degree of BNIS to assess the allocation of SENHs based on the Chome-level data in the ward area of Tokyo.

SENHs offer intensive nursing care for the elderly who require care levels 3–5 (ER3-5, for short), especially the bedridden. In Japan, the elderly who require healthcare are classified into 7 levels: support 1, support 2, care 1, care 2, care 3, care 4, care 5, based on health status and required nursing time. The higher the level, the higher the requirement for nursing is. In principle, only the elderly at levels of care 3, 4, and 5 (recognized by the government) will be allowed to use SENHs. It is referred as the “last habitat” to safeguard better health in the later years of life. The ER3-5 tend to choose SENHs that are close to their homes with familiar environments. This is largely due to their relatively difficult living conditions and lower levels of physical fitness ER3-5. The allocation of SENHs at the sphere of welfare, ward, and sphere of daily life in Tokyo (organized by departments) are generalized into two main methods for estimating the beds-to-elderly population ratio (BPR method) and the population of the ER3-5. The BPR method is applied in one ward and the sphere of welfare (several contiguous wards) [3], and the population of the ER3-5 is counted at the sphere of daily life (a group of Chome) [4]. These two methods combined create a top-down process defining the three levels of the sphere of welfare and ward, as well as the sphere of daily life, but lacks estimation of the BPR at the Chome (the smallest administrative unit in Japan). It is one origin of this study that even the lowest level of the sphere of daily life in programs is still too large and dissimilar to assess the allocation of SENHs in practice (spheres of daily life were delimited by the distribution of the population with homogeneous socioeconomic or demographic characteristics). In this paper, the Chome is the basic spatial unit to calculate the BNIS and map the distribution of the degree of BNIS.

BNIS represents the required number of beds for SENHs (i.e., the population of the ER3-5 that needs to use SENHs but have difficulty acquiring nursing care). The distribution of the degree of BNIS (the distribution of the population of the ER3-5 that needs to use SENHs) will be founded from the Chome with consistent BNIS in a certain range. This spatial pattern is not restricted by the administrative boundary of the Chome, since its size is smaller than the catchment area of SENHs. It implies that new locations of SENHs are not only limited in the Chome with high BNIS, but also neighboring areas within the catchment area. Assessing the allocation of SENHs at the level of the Chome can be regarded as supplementary, and SENHs would be better placed where the degree of BNIS are the highest. Furthermore, the degree of BNIS is determined by the population of the ER3-5 lacking access to SENHs, which consists of two groups: the population of the ER3-5 that exceeds the capacity of SENHs within the catchment area and the population of the ER3-5 outside this area. The higher BNIS is, the more important it is for departments and social welfare corporations to construct SENHs. Related open data on the ER3-5 and their socioeconomic and demographic characteristics at the Chome level have provided the possibility of calculating BNIS at a smaller spatial unit.

In the BPR method, the elderly population is used as the population demand in the denominator. Although the elderly population can be replaced by the population of the ER3-5 to improve accuracy of the BPR [5], two implicit shortcomings are present with the BPR method: first, it assumes that the ER3-5 within the same sphere of welfare or ward have equal access to SENHs, which signifies the distance between SENHs and the ER3-5 (convenience for acquiring nursing care) is not fully considered; secondly, consideration for potential demand of the ER3-5 to use SENHs is insufficient, because the needs of ER3-5 subgroups differ in terms of socioeconomic and demographic characteristics. Even departments of the ward have used partial characteristics of the ER3-5 (including family structure, dwelling situation, and outside activity, etc.) to evaluate the allocation of SENHs through paper investigations or onsite visits [6]. However, it may capture only part of the data from the ER3-5, due to the limitation of field surveys. Meanwhile, it is difficult for departments to quantify and apply these data in estimating the BPR simply by the BPR method. It will be helpful to develop methods that can adopt the parameter of distance between SENHs and the ER3-5, and optimize the population of the ER3-5 by considering potential demand.

Many pieces of literature have suggested using or enhancing the two-step floating catchment area method to add the parameter of distance between supply and demand in estimating a reasonable ratio of the capacity of facility and targeted population [7,8,9,10]. The premise behind the “two-step” in this method is that the spatial unit for estimating BPR is generally larger than the catchment areas of targeted facilities. In this paper, we introduce the original two-step floating catchment area method to estimate BPR at both spatial units that are larger and smaller than the catchment area of SENHs (abbreviated “FCA”). FCA addresses the shortcomings of the BPR method, and can be applied as a supplemental tool for estimating the BPR at the sphere of welfare, ward, and the level of Chome as well. Catchment area in the FCA represents a service zone with a certain distance. To determine the catchment area of a facility, many studies compute coverage areas by setting certain travel times based on the road network data [11,12,13,14]; and several studies use different distances to generate service zones according to characteristics of specific facilities to designate the catchment area [15,16,17]. Most studies considered the catchment area according to the types and capacities of facilities, and the density of population to be served, etc. In this paper, the catchment area is further generated by various distances according to the standard of average population served by the SENHs and capacity, in which the capacity of SENHs is denoted by beds.

For the population of the ER3-5 in the FCA, the quantity is not only influenced by health status but also their socioeconomic and demographic characteristics [18,19]. It has been a significant aspect to further enhance the application of the FCA in the construction of aged care facilities. The core is how to convert socioeconomic and demographic characteristics data into coefficients to improve the parameter of elderly population [16,20]. This is a benefit for satisfying the elderly needing help. Meanwhile, there are close, internal connections among variables in real socioeconomic and demographic characteristics data of the population [21]. A simple combination of variables, ignoring correlations in calculating coefficients is inaccurate [22]. Hence, this paper presents a multivariate linear model to quantify the impact of variables on the population of ER3-5 needing SENHs. The result is a coefficient of the potential demand of the ER3-5. This model is a technique to improve the population of the ER3-5 in the FCA.

The introduced FCA was first proposed to replace original availability measures (such as the gravity model) for facilities, etc., which usually considers the distance between supply and demand with distance decay [23,24,25,26]. With the development of method study, the majority of current researches focus on enhancing the FCA from the perspective of defining the parameter of catchment for facilities, because the original FCA ignores differences of accessibility with the catchment [11,12,13,14,15,16,17]. However, discussions for improving the measures to the other key parameter of the demand in the FCA are relatively less [16,20]. This is significant to further enhance the accuracy and operability of the FCA in real-world applications, especially for assessing the allocation of healthcare facilities. Therefore, this study attempts to further develop the existing FCA to assess the allocation of SENHs from demand aspect, and illustrate the solution to the practical problem in Tokyo via the corresponding introduction and computation of coefficient.

Building on the proposed research goals and prior research, this paper makes the following contribution: It introduces a BNIS and maps the distribution of the degree of BNIS to assess the allocation of SENHs based on a parameter-improved FCA at the level of the Chome. We adopt coefficients of various distances and potential demand of the ER3-5 into the original FCA to improve the accuracy for assessing the allocation of SENHs. Among them, the coefficient of distances is determined using the standard of the average population served by the SENHs and beds, the coefficient of the potential demand of the ER3-5 is computed via building a multivariate linear model. This assessment may help departments determine the priority for approving locations of SENHs more accurately with a limited budget in short-term construction.

The paper is structured as follows. Section 2 describes the dataset of spatial units, the ER3-5, SENHs, and methodology. Section 3 presents the coefficient of the potential demand of the ER3-5 to modify population of ER3-5 that needs to use SENHs. It then demonstrates estimates by the BPR method, FCA, PI-FCA, and the comparative analysis between the FCA and PI-FCA. Lastly, it provides the distribution of the degree of BNIS for allocation assessment of SENHs. Finally, Section 4 summarizes the conclusions of this paper and briefly discusses contributions and future directions of this research.

## 2. Materials and Methods

### 2.1. Study Area and Data Sources

**Figure 1:**
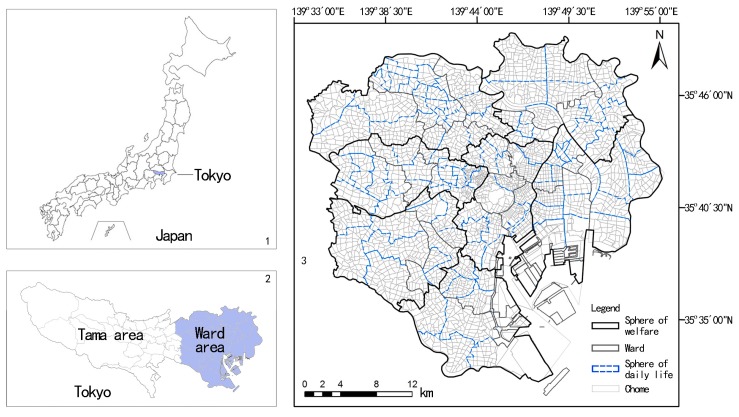
Spatial units within the ward area of Tokyo.

**Figure 2:**
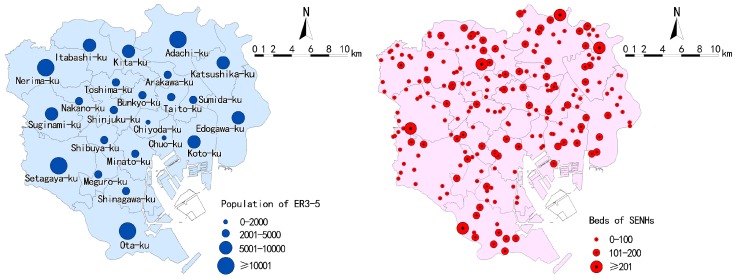
Distributions of the population of the elderly who require care levels 3–5 (ER3-5) ER3-5 and beds.

The ward area of Tokyo covers 23 wards. Distributions of the sphere of welfare and sphere of daily life (designated by programs), ward, and the Chome are shown in Figure 1. There are a total of seven spheres of welfare, 124 spheres of daily life, and more than 3000 Chome. The average size of a Chome is 10–20 hectares. There are 1,927,765 elderly populations living in the ward area, in which the population of the ER3-5 is 130,671 (the distributions at the ward are shown in Figure 2). In general, the population of the ER3-5 spatially increases from central to peripheral wards.

Unlike the present BPR method in programs, this research employs the Chome as the census tracts of the ER3-5, in which centroids of the Chome are used to represent the location of the ER3-5. On the condition that the distribution of the ER3-5 is directly linked to their age composition (according to the statistical analysis of population of ER3-5 for ages), we relocate population of ER3-5 at the Chome level according to the proportion of elderly population at each age group is equal to the proportion of ER3-5 at each age group between the Chome and ward. The population of the ER3-5 at Chome is obtained by summing the population of the ER3-5 at each age group. In the above process, the data of population of the ER3-5 for all ages is collected from the Status Report on Long-term Care Insurance Plan in 2015 (monthly), which was published on the official website of the Long-term Care Insurance Division, Bureau of Social Welfare and Public Health (http://www.fukushihoken.metro. tokyo.jp). The elderly population data of each age group of ward and Chome are acquired from the Social-Economic Statistics (CSV) of the Department of Statistics, Ministry of Internal Affairs of Japan (https://www.e-stat.go.jp).

SENHs data (shape file) of the ward area in 2015 was downloaded and filtered from the official information of land and resources website, which was released by the Ministry of Land, Infrastructure, Transport and Tourism (http://nrb-www.mlit.go.jp/ksj/index.html). There are 220 SENHs (totaling 18,915 beds) in the ward area, and their distributions are shown in Figure 2. The 2010 elderly population and related socioeconomic and demographic data at the Chome are based on the Social-Economic Statistics (CSV) of the Chome as well. These data are analyzed and visualized in Geographic Information System (GIS) (ArcGIS 10.2, Environmental System Research Institute, Inc. (ESRI), Redlands, CA, USA) and Statistical Product and Service Solutions (SPSS) software (SPSS 23.0, International Business Machines Corporation (IBM), New York, NY, USA).

### 2.2. Methods

**Figure 3:**
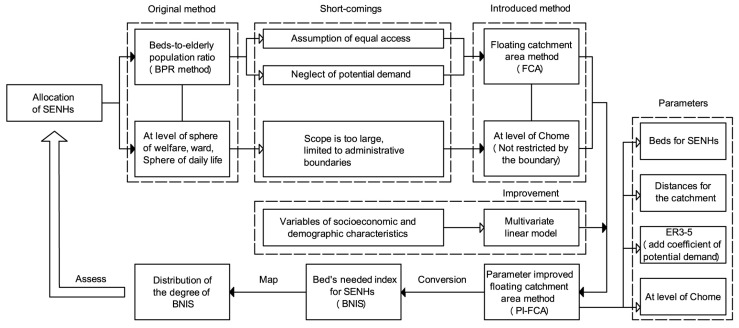
Framework chart.

We synthesize the previously mentioned shortcomings in the original method (BPR method) of Tokyo, to address the need to enhance the rationality of phased construction of SENHs. We introduce four basic steps to illustrate our methods, variables (parameters) and analytical logics about the allocation assessment of SENHs. In the following, we state each of these steps to reach the ultimate goal of assessing the allocation of SENHs. Firstly, a FCA is introduced to consider the parameter of distance between SENHs and the ER3-5ER3-5, and we increase the assessment for allocation of SENHs at the level of the Chome as well. Secondly, we further improve the original FCA by applying the coefficient of potential demand of the ER3-5 to original parameters. It is based on the enhancements for measuring the parameter of demand in the FCA are relative less. A multivariate linear model is established to quantify the impact of variables from socioeconomic and demographic characteristics to the population of ER3-5 that need to use SENHs. Weights of the population of ER3-5 are computed at each sphere of welfare, ward, and the Chome. Significant factors in the multivariate linear model are obtained using the factor analysis tool in the SPSS. After applying PI-FCA at the sphere of welfare, ward, and the Chome, we compare the results of the BPR method, FCA, and PI-FCA, to illustrate the merits of the PI-FCA. Thirdly, for full coverage of study area, a BNIS is proposed and computed via the BPR, which is calculated by the PI-FCA. The BNIS consists of two groups of ER3-5: the population of the ER3-5 that exceeds beds of SENHs within the catchment area and the population of the ER3-5 outside the catchment area. Finally, we map the distribution of the degree of BNIS in a GIS environment using the degree of BNIS from 1 (least important) to 5 (most important), to assess the allocation of SENHs. The framework for the proposed model in this paper is shown in Figure 3.

#### 2.2.1. A Parameter-Improved FCA

To differentiate between catchment areas, the buffer analyst in the GIS is used to generate multiple service zones according to the standard of the average population served by SENHs and beds [20]. It is implemented to improve the distance between SENHs and the ER3-5.

For each SENHs (j), in this paper, we define its catchment area as three zones (zones 1, 2, and 3) according to recommended standards of the average population served by SENHs that should be available within a certain territorial scope of 20,000 residents in Japan [27]. Hence, the basic catchment area of SENHs is defined as a circular area with a distance of 800 m based on the population density in the ward area of Tokyo. In addition, the catchment area of each SENHs is influenced by its beds. The current SENHs are dominated by small-capacity facilities in Tokyo, in which the number of beds are less than 100. SENHs with more than 100 beds can serve the ER3-5 that are more than 800 m away in theory. The distances between SENHs and the ER3-5 with 100–200 beds and more than 200 beds are 1000 and 1200 m, respectively. The three zones, generated by three distances of 800, 1000, and 1200 m, are zones 1, 2, and 3, respectively. Each SENHs is searched by the ER3-5 in a corresponding catchment area, and the original BPR within the catchment area in the FCA can be converted from Equation (1) to Equation (2):

(1) $${\mathrm{BPR}}_{j}=\frac{D_{j}}{\sum_{k\in \left(d_{kj}\leq d_{o}\right)}E_{k}}$$

(2) $${\mathrm{BPR}}_{j}=\frac{D_{j}}{\sum_{k\in \left\{d_{kj}\leq d_{o}\left[d_{o}\in \left(d_{1},d_{2},d_{3}\right)\right]\right\}}E_{k}}$$

where $D_{j}$ is the beds at SENHs *j*, $d_{kj}$ is the distance between SENHs *j* and subdistrict *k* (the centroids of the Chome), $d_{o}$ is the threshold service distance between SENHs and the ER3-5, and $E_{k}$ is the population of the ER3-5 in sub-district *k*. Specifically, $d_{o}$ has three values of $d_{1}$, $d_{2}$, and $d_{3}$*.*

In order to quantify the potential demand of ER3-5 for SENHs, we apply a coefficient of potential demand of the ER3-5 ($C_{pd}$) into the FCA. It represents the weight of the population of the ER3-5 in varied conditions that need to use SENHs in the catchment area. $C_{pd}$ is calculated by a multivariate linear model and its special process will be analyzed in Section 2.2.2. The BPR within the catchment area after improving two parameters is as follows:

(3) $${\mathrm{BPR}}_{j}=\frac{D_{j}}{\sum_{k\in \left\{d_{kj}\leq d_{o}\left[d_{o}\in \left(d_{1},d_{2},d_{3}\right)\right]\right\}}E_{k}C_{pd}}$$

To estimate the total BPR within the catchment area in wards based on the Chome-level data, for each ward (*i*), we search all locations of Chome in the corresponding catchment area of SENHs (*j*). We assume the ER3-5 outside the catchment area have limited access to services. The total *BPR* ($TBPR_{i}$) of each ward can be calculated by adding all BPR within catchment areas using Equation (4). For a Chome that is smaller than the catchment area of SENHs, the BPR within the catchment area is determined by the population of the ER3-5 and available beds. An assumption is made that beds that can be used by the ER3-5 at each Chome in the same catchment area are equal. Thus, the *BPR* within the catchment area of the Chome ($BPR_{k}$) should divide the number of Chome ($N_{C}$) in the catchment area using Equation (5).

(4) $${\mathrm{TBPR}}_{i}=\sum_{j\in \left\{d_{ij}\leq d_{o}\left[d_{o}\in \left(d_{1},d_{2},d_{3}\right)\right]\right\}}BPR_{j}$$

(5) $$BPR_{k}=\frac{D_{j}}{E_{k\in \left\{d_{kj}\leq d_{o}\left[d_{o}\in \left(d_{1},d_{2},d_{3}\right)\right]\right\}}C_{pd}N_{C}}$$

#### 2.2.2. A Multivariate Linear Model

$C_{pd}$, the quantitative potential demand of the ER3-5 using SENHs, vary due to different socioeconomic and demographic characteristics of the ER3-5 at the sphere of welfare, ward, and the Chome. We assume that, besides the health status, whether the ER3-5 need to use SENHs is influenced by their socioeconomic and demographic characteristics. The possibility of using SENHs is *P* and the factor of the socioeconomic and demographic characteristics is *V*:

(6) P=f(V)

After summarizing the variables of the socioeconomic and demographic characteristics, we select significant categories from the variables based on correlation analysis between existing beds and each percentage of categories in variables at the ward.

The more beds in the ward, the greater the need to use SENHs. The *V* is a linear function that explains significant categories through a progressive linear regression analysis between existing beds and the percentages of significant categories at the ward (*R*^2^ = 0.899, *Sig*. = 0.000). It indicates that these selected significant categories have a relative direct impact on ER3-5 that need to use SENHs. Their relationship can be explained by:

(7) $$V=\beta_{0}+\beta_{1}X_{1}+\beta_{2}X_{2}+\ldots +\beta_{n}X_{n}$$

where *X* is the weight coefficient that denotes the influence of significant categories on SENHs by the ER3-5. β is constant, and $\beta_{0}$ is 0 based on the analyzed result. Let n denote the number of significant categories.

The results of linear regression analysis proved the linear relationship between the significant categories and ER3-5 that need to use SENHs. As the premise of quantifying the impact of each significant category to the ER3-5, we adopt further factor analysis technology to decide the weight of each significant category and apply this to a multivariate linear model to organize the results of $C_{pd}$. In determining the weight of each significant category (variable), the underlying assumption has been made by some previous studies was an equal weight for each variable [24]. However, variables, especially for socioeconomic and demographic characteristics, are usually correlated and a simple combination of variables ignoring correlations is inaccurate [22,28]. There are specific advantages to using factor analysis in this study: many variables are consolidated into a few key variables for easy interpretation, and the weight for each key variable can be relatively accurately calculated to produce $C_{pd}$ for each ward and Chome [29]. Next, the main equations in the factor analysis, reorganized according to [30,31], are as follows:

(8) DPij=Lpc/Epc^(1/2)

(9) $$CR_{pc}=\sum_{m=1}\left(DP_{m1}V_{pcm}\right)/\sum_{m=1}\left(V_{pcm}\right)$$

where $DP_{ij}$ is the determination principle coefficient of each used category in the linear combination. $L_{pc}$ is the principal component load, and $E_{pc}$ is the principal component eigenvalues. In Equation (9), $CR_{pc}$ is the principal component contribution rate of each used category. $V_{pc}$ is the principal component variance. Among these, $L_{pc}$, $E_{pc}$, and $V_{pc}$ are based on the results of factor analysis in SPSS.

To calculate the correction coefficients at each sphere of welfare, ward, and Chome (V), we adopt the coefficient $CRN_{pc}$ which can be obtained by normalization of $CR_{pc}$ and the percentage of the population of the ER3-5 that belongs to each significant category ($P_{np}$). The multivariate linear model is Equation (10), and further results of $C_{pd}$ at the sphere of welfare, ward, or Chome are calculated through *V* divided by their averages.

(10) $$\{\begin{array}{c} V_{1}=P_{11}CRN_{pc1}+P_{12}CRN_{pc2}+\cdots +P_{1p}CRN_{pcp}\\ V_{2}=P_{21}CRN_{pc1}+P_{22}CRN_{pc2}+\cdots +P_{2p}CRN_{pcp}\\ \cdots \\ V_{n}=P_{n1}CRN_{pc1}+P_{n2}CRN_{pc2}+\cdots +P_{np}CRN_{pcp} \end{array}$$

The PI-FCA is used to calculate BNIS and help departments determine the priority for approving locations of SENHs more accurately. The first advantage of the PI-FCA is that it is the method, set catchment area based on the standard of average population served by SENHs and beds, to meet the needs to focus on the quality of SENHs service and single-reference distance (equity of planning). Secondly, it considers the potential demand of the ER3-5 by adopting $C_{pd}$ using a multivariate linear model that improves upon simply using the elderly population as the demand size for SENHs. The result of the PI-FCA is a critical step to calculating BNIS and an in-depth method of BPR evaluating the overall allocation of SENHs. It is easy to implement and visualize in GIS environments.

## 3. Results

### 3.1. Coefficient of Potential Demand of ER3-5 ($C_{pd}$)

Demands of the ER3-5 vary between subgroups with different socioeconomic and demographic characteristics. We summarize it as family environment and dwelling condition, according to the data from Social-Economic Statistics (CSV) in Tokyo. Family environment mainly contains four variables of sex, age, education level, and family structure, which include 14 categories. The dwelling condition includes three variables: house type, house floor, and living space, which include 15 categories. Data for these categories are collected and expressed in terms of percentage. Results of correlation analysis between existing beds and each percentage of categories at ward are shown in Table 1. The ER3-5 of age groups 65–84, living in family of elderly group, public housing (constructed by municipality), 1–5 floors, and 70–149 m^2^, have a higher tendency to the needs for SENHs.

Although the correlation analysis and linear regression analysis has selected relatively significant categories that may influence the needs for SENHs and examined their linear relationships, respectively, the factor analysis still needs to be adopted to quantify the weight for each significant category. Factor analysis is a mathematical method with linear techniques to analyze the dimensionality of data in multivariate problems [32]. Significant categories should meet the test of KMO (Kaiser–Meyer–Olkin) and Bartlett’s test is the primary condition for using factor analysis. The KMO index with 0.6 suggested as the minimum value for a good factor analysis and Bartlett’s test of sphericity should be significant (*p* < 0.05) [33]. In this study, it indicates that significant categories should be correlated with the number of beds and there exists high interconnection between significant categories [34]. A test of 10 significant categories shows that data from significant categories are suitable for factor analysis (the KMO measure of sampling adequacy is 0.710 and the significance of Bartlett’s test of sphericity is 0.000). Weights for significant categories are calculated via the parameters of $L_{pc}$, $E_{pc}$, and $V_{pc}$ from factor analysis, as (in order), 0.099, 0.085, 0.092, 0.119, 0.134, 0.065, 0.075, 0.078, 0.134, and 0.118. Hence, the $C_{pd}$ can be computed by multivariate linear models combined with percentages of significant categories at ward and Chome. Values of $C_{pd}$ at the 23 wards are shown in Table 2. The original population of ER3-5 in each ward is modified by $C_{pd}$ to different degrees. The $C_{pd}$ plays a vital role in measuring the population of the ER3-5 that need to use SENHs. Modified populations of the ER3-5 by $C_{pd}$ can be further applied in the PI-FCA to enhance estimates.

### 3.2. Estimates by BPR Method, FCA, and PI-FCA at Spheres of Welfare and Ward

**Figure 4:**
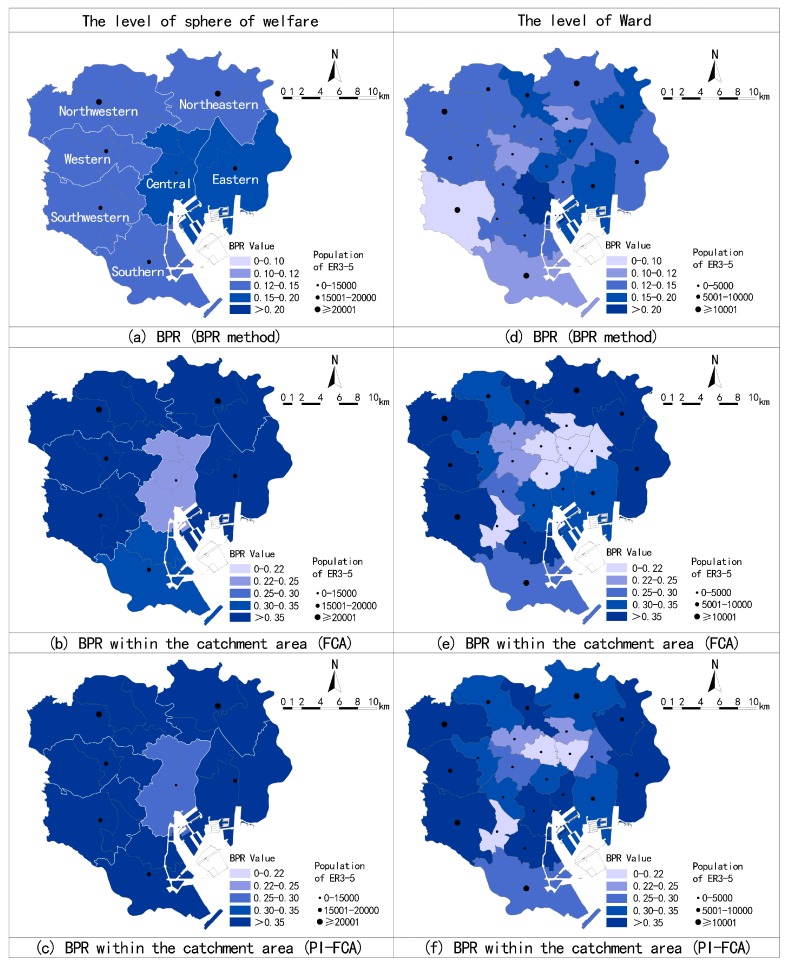
Estimates by the beds-to-elderly population ratio (BPR) method, floating catchment area method (FCA), and parameter-improved floating catchment area method (PI-FCA) at sphere of welfare (**a**–**c**), and ward (**d**–**f**).

Results of applying the FCA and PI-FCA at the sphere of welfare and ward can supplementary to present assessment method (BPR method), and embody the merits of the PI-FCA. Estimates by the BPR method, FCA, and PI-FCA are shown in Figure 4. Overall, estimates by the BPR method are significantly less than the BPR within the catchment area by the FCA and PI-FCA. It reveals that a large population of the ER3-5 is outside the maximum service zones of SENHs in the 23 wards. Identified distribution at the shortage sphere of welfare and ward are various from methods.

At the level of sphere of welfare, there is a higher BPR in central and eastern wards, but the BPR within the catchment area of central wards is relatively low by FCA. The issue of limited beds is eased to some degree after parameter improvement. It illustrates that the potential demand from the ER3-5 in the catchment area of central wards is reduced. In general, the supplied beds in catchment area are relatively less, although the total beds in the central wards are high. At the level of ward, the ward with the lowest BPR within the catchment area is the Setagaya-ku, and Arakawa-ku, Shinjuku-ku, and Ota-ku are less. However, wards with low BPR within the catchment area are the Arakawa-ku, Bunkyo-ku, Taito-ku, Sumida-ku, and Chiyoda-ku in the central wards and the Meguro-ku in the southwestern wards. The PI-FCA makes certain modifications to the results of the FCA. It diminishes shortage wards, which are concentrated in Bunkyo-ku, Taito-ku, and Meguro-ku, and simultaneously increases the BPR within the catchment area of Chuo-ku and Minato-ku. It reflects that the socioeconomic and demographic characteristics of the ER3-5 vary between wards, especially in the central wards.

**Figure 5:**
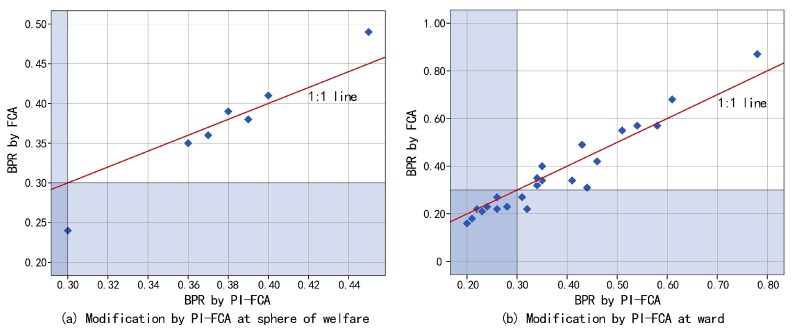
Comparative analysis to illustrate modification of the BPR by the PI-FCA: (**a**) sphere of welfare, and (**b**) ward.

Comparisons of the BPR by the FCA and PI-FCA in each sphere of welfare and ward are shown in Figure 5. Overall, $C_{pd}$ modifies the BPR within the catchment area of each sphere of welfare and ward to different degrees. The PI-FCA increases the results of the FCA in most cases, especially for the shortage spheres of welfare and wards (taking the BPR within the catchment area of 0.3 as the threshold value). It implies that the FCA underestimates the results without considering the potential demand of the ER3-5 in more cases in estimating the BPR within the catchment area of the ward. As Figure 5 shows, more data points of the ward are below the 1:1 line.

### 3.3. Estimates at Chome and Distribution of the Degree of BNIS

**Figure 6:**
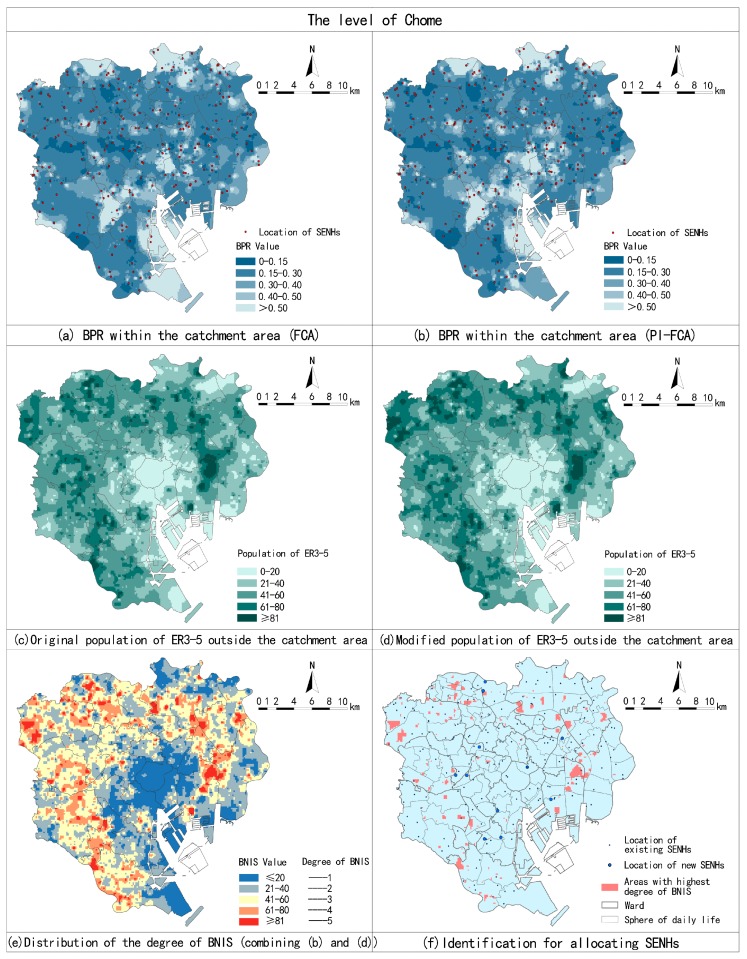
Estimates by the FCA and PI-FCA (**a**,**b**), population of the ER3-5 before and after applying $C_{pd}$ (**c**,**d**), distribution of the degree of BNIS, and identification for allocating SENHs (**e**,**f**).

The BPR within the catchment area at the sphere of welfare and ward are based on administrative boundaries. The estimation at the Chome level is supplementary to the BPR method. Estimates by the FCA and PI-FCA are processed via spatial interpolation techniques in GIS, as shown in Figure 6a,b. The results show they generate similar overall spatial patterns yet differ in specific distributions.

**Figure 7:**
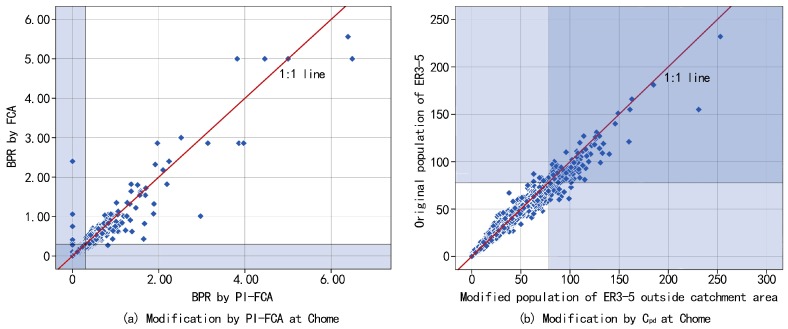
Comparative analysis to illustrate modification of the BPR by the PI-FCA (**a**), and modification of the population of the ER3-5 by $C_{pd}$ (**b**).

The BPR within the catchment area of the Chome is modified to some degree, especially for shortage Chome. The PI-FCA decreases the results of the FCA in most cases, which illustrates that FCA overestimates the BPR (Figure 7a). However, the number of Chome with the lowest BPR within the catchment area is increased. As mentioned above, BNIS is the required number of beds for SENHs, which represented the population of the ER3-5 that needs SENHs but have difficulty acquiring nursing care. It contains the population of the ER3-5 within and outside the catchment area. Therefore, we convert the BPR within the catchment area by the PI-FCA into the population of the ER3-5 lacking access to SENHs, based on the total population of the ER3-5 minus available beds. We measure the ER3-5 outside the catchment area at Chome due to the results of PI-FCA, to evaluate the BPR within the catchment area. The distributions of the original population of the ER3-5 and modified population of the ER3-5 by $C_{pd}$ outside the catchment area are shown in Figure 6c,d. Overall, the number of shortage Chome (the population of the ER3-5 in each Chome the outside catchment area that is greater than or equal to 81) is increased with more data points of Chome below the 1:1 line (Figure 7b), to be used as one part of BNIS. The distribution of the degree of BNIS in the 23 wards is shown in Figure 6e, by combining the population of the ER3-5 without services within the catchment area and the population of ER3-5 outside the catchment area. The degree of BNIS is 5 (the highest degree of BNIS), which represents that most of the population of the ER3-5 needs SENHs but without reasonable service distances. These areas have the most urgent need to allocate SENHs. Identification of SENHs allocation is shown in Figure 6f. Areas with the highest degree of BNIS are distributed dispersedly across the entire ward area where the concentrations are mainly located in peripheral wards. Proposals for new location of SNHEs in these areas would be considered and approved in priority.

**Figure 8:**
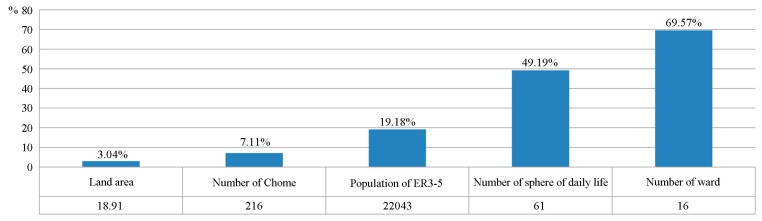
Related statistics for the highest degree of BNIS.

In general, areas covered by the highest degree of BNIS have a total of 18.91 km^2^ (involving 216 Chome). This area contains 22043 ER3-5 without adequate access to SENHs. Meanwhile, there are 61 spheres of daily life, and 16 wards have Chome with the highest degree of BNIS (Figure 8). These statistics may help departments understand the overall construction of SENHs. On the other hand, Figure 8 shows there are 11 wards that have determined the location of new SENHs. However, the others planned to increase SENHs with no decision on the location, according to the programs of each ward. Estimating BNIS based on the PI-FCA and improving the population of the ER3-5 can be used not only to provide a basis for determining the priority for approving SENHs locations, but also for allocating new SENHs. It is helpful to implement the approval and planning of practical SENHs.

## 4. Discussion

Using this research to address, there is need to enhance the rationality of phased construction of SENHs. The number of beds at SENHs is far less than the elderly population in need, which has been a prominent problem. This assessment may help social welfare and public health departments determine the priority for approving locations of SENHs and planning new SENHs more accurately with a limited budget in short-term construction. As a critical step to calculating BNIS, there are advantages of applying the PI-FCA in SENHs programs in Tokyo. For example, in practice, the consideration of the distance between SENHs and the ER3-5 meet the need to focus on the quality of SENHs service and equity of planning. In addition, the proposed potential demand of ER3-5 is derived from the satisfaction of the ER3-5’s actual demand. These two parameters in the PI-FCA can be easily computed using GIS and SPSS. Introducing BNIS into existing practices of approving and planning new SENHs would help departments allocate aged-care resources to the most needy areas. This idea can be used in the other public healthcare facilities. Additionally, the BNIS can be recalculated using changing data to regularly review programs.

Several issues remain for further study. First, we apply the straight-line distance instead of road network distance, due to the catchment area being mainly determined by allocation standard. This ignores the actual capacity of the road to some degree and results certain assessment differences. Second, this paper focused on calculating BNIS, which is mainly used for guiding construction approval, but does not suggest concrete location optimizations. Future research will propose location optimizations based on present assessments, which combine with multiple demands for building or adjusting SENHs.

## 5. Conclusions

This paper calculates the BNIS based on the Chome-level data in the ward area of Tokyo. An FCA is adopted, to increase the consideration of the distance between SENHs and the ER3-5, and optimize the measurement of the population of the ER3-5 that needs SENHs. Improvements for the FCA are further developed, to more correctly identify distances between SENHs and the ER3-5, and potential demand of the ER3-5, by adding coefficients of various distances of the catchment area (using the standard of average population served by SENHs and beds) and potential demand of the ER3-5 (using a multivariate linear model). At the level of spheres of welfare and wards, comparative results of the BPR method and FCA show that a large population of the ER3-5 is outside the catchment area of SENHs. Wards with relatively lower BPR are distributed in periphery areas, but wards with relatively lower BPR within the catchment area are concentrated in central areas. Meanwhile, the PI-FCA identifies a more concentric spatial distribution of lower BPR within the catchment area of the ward. The PI-FCA estimates improved results, compared with the FCA. At the level of Chome, results of the FCA and PI-FCA have similar spatial patterns, but the PI-FCA identifies larger areas with the lowest BPR within the catchment area. Based on population of the ER3-5 without services within the catchment area, we further map the distribution of the degree of BNIS in the ward area of Tokyo, by combining the population of the ER3-5 that exceeds beds within the catchment area and the population of ER3-5 outside the catchment area. The BNIS not only helps departments the find most important areas for allocating SENHs, but also increases the overall understanding of the construction of SENHs, through related statistics for the highest degree of BNIS.

## Figures and Tables

**Table 1:** Correlation analysis between existing beds and each percentage of categories at ward.

Aspects	Variables	Categories	Correlation Coefficients
Family environment	Gender	Male	0.378
		Female	−0.378
	Age	65–70	0.632 **
		70–75	0.601 **
		75–80	0.514 *
		80–85	0.703 **
		85–90	−0.249
		Over 90	−0.668
	Education level	College or above	−0.468
		Technical school	0.190
		High school or below	0.410
	Family structure	Live alone	−0.488
		Elderly group	0.505 *
		Live with others	0.345
Dwelling condition	House type	Private housing	0.291
		Private leasing	−0.334
		Public housing	0.430 *
		Issued housing	−0.456
		Others	−0.282
	House floor	1–2 floor	0.509 *
		3–5 floor	0.550 **
		6–11 floor	−0.554
		>11 floor	−0.487
	Living space	≤29 m^2^	−0.453
		30–49m^2^	−0.238
		50–69 m^2^	0.189
		70–99 m^2^	0.508 *
		100–149 m^2^	0.495 *
		≥150 m^2^	0.215

Note: ** denotes correlation is significant at level of 0.01 (two tails); * denotes correlation is significant at level of 0.05 (two tails).

**Table 2:** $C_{pd}$ value at each ward.

Name of Ward	Population of the Elderly Who Require Care Levels 3–5 (-)	$C_{pd}$ Value	Name of Ward	Population of the ER3-5	$C_{pd}$ Value
Chiyoda-ku	817	0.68	Shibuya-ku	2641	0.88
Chuo-ku	1636	0.71	Nakano-ku	4176	0.96
Minato-ku	3126	0.82	Suginami-ku	7757	1.05
Shinjuku-ku	4245	0.83	Toshima-ku	4210	0.94
Bunkyo-ku	3010	0.85	Kita-ku	5616	0.98
Taito-ku	2887	0.81	Arakawa-ku	3053	0.92
Sumida-ku	3667	0.85	Itabashi-ku	8003	1.03
Koto-ku	6055	0.94	Nerima-ku	10,812	1.15
Shinagawa-ku	4344	0.91	Adachi-ku	11,572	1.14
Meguro-ku	3859	1.02	Katsushika-ku	7705	1.11
Ota-ku	10,712	1.02	Edogawa-ku	7464	1.07
Setagaya-ku	13,304	1.11			
